# Exploring the binding pattern between pepsin and deferasirox using detailed experimental and computer simulation methods

**DOI:** 10.1039/c8ra07993e

**Published:** 2018-11-05

**Authors:** Ji Yang, Qiaohong Du, Na Gan, Yongkuan Chen, Liu Yang, Zhihua Liu, Hui Zhao, Qiaomei Sun, Hui Li

**Affiliations:** School of Chemical Engineering, Sichuan University Chengdu Sichuan 610065 China lihuilab@sina.com +86 028 85401207 +86 026 85405220; R&D Center of China Tobacco Yunnan Industrial Co., Ltd. Kunming 650231 China cyk1966@163.com

## Abstract

Steady-state fluorescence spectroscopy indicated that a ground state complex was formed between deferasirox (DFX) and pepsin. The binding parameters and thermodynamic parameters of pepsin–DFX complex formation suggested the presence of only one high affinity binding site in the binding process of DFX and pepsin and that the binding process was hydrogen bond dominated. According to the MD simulation optimal pepsin–DFX binding model analysis, the binding force between DFX and pepsin was mainly hydrogen bonding, and the hydrophobic interaction was supplemented. Synchronous fluorescence spectroscopy and 3D fluorescence spectroscopy indicated that the binding of DFX to pepsin had minor effect on the protein structure and function. Circular dichroism spectra showed that DFX had no significant effect on the main secondary structure of pepsin. MD analysis also showed that DFX did not affect the looseness of pepsin and the overall secondary structure, but it affected the amino acid residue sequence Leu48-Ala49-Cys50-Ser51-Asp52. Pepsin enzyme activity test showed that the addition of DFX had a slight enhancement effect on the activity of pepsin. Combined with the MD results, DFX bound to pepsin and was closer to the pepsin active site Asp-215, which may affect the electrical environment of Asp-215 residues and enhance the activity of pepsin.

## Abstract 2

Investigation on the binding properties of deferasirox to pepsin.

## Graphical abstract

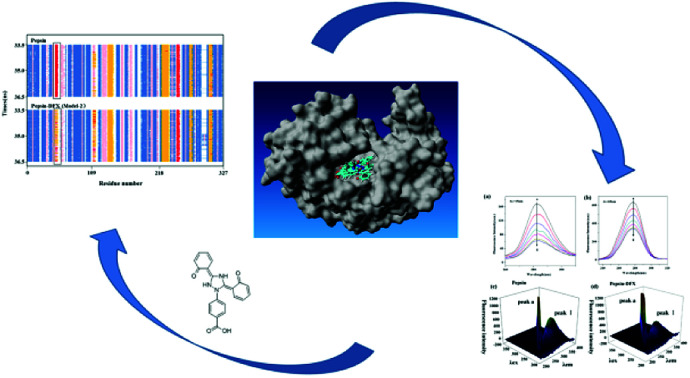

## 1 Introduction

**Fig. 1:**
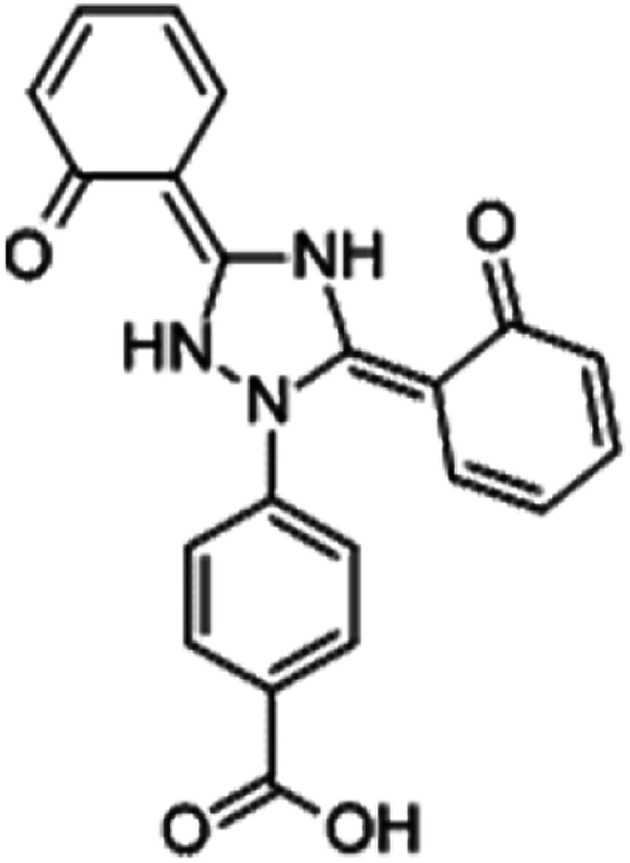
Structure of deferasirox.

The drug deferasirox (DFX) (Fig. 1) is the first oral iron-loading agent approved by the US Food and Drug Administration (FDA) for the treatment of chronic iron overload. DFX has shown good application prospects with its anti-fungal, anti-cell proliferation, anti-malarial, anti-oxidative stress damage, anti-cytotoxicity-induced apoptosis, and other pharmacological effects.^1,2^ The most common side effect of oral iron sulfate (DFX) is gastrointestinal upset, including abdominal pain, diarrhea, nausea, and vomiting. However, very few patients will have severe symptoms of gastrointestinal bleeding.^3^ After oral administration of the drug to the stomach through the mouth, the drug easily combines with the important digestive protease in the stomach, *i.e.*, pepsin, thereby affecting the activity of pepsin, causing abdominal pain, nausea, vomiting, and other adverse symptoms.^4^ Pepsin is the product activated by pepsinogen and is secreted by the chief cell of the gastric gland. It is widely found in the gastric juice of mammals and hydrolyzes proteins in an acidic environment.^5,6^ The catalytically active site of pepsin consists of two Asp, namely, Asp-32 and Asp-215. One of these two amino acids is protonated to activate pepsin, whereas the other is deprotonated to activate pepsin.^7^ When the balance of invasive factors and protective factors of gastric mucosa is destroyed, pepsin can cause damage to the gastric mucosa, which leads to diseases like ulcers. Therefore, studying the binding interaction between the drug and pepsin can provide a scientific basis for the treatment of gastric diseases induced by drug.^8,9^ Based on the abovementioned discussion, it is necessary to study the interaction between DFX and pepsin. Moreover, as the basic unit of most life activities, protein is the most important component of the body's cells. It occupies most of the weight composition of the living body. It has many functions, such as regulating intracellular material transport, signal transduction, metabolism, catalysis and modification, and is the main performer of life activities. Studying the interactions between drugs and proteins, such as binding mechanisms, binding sites, binding constants, and effects on protein structure and function, can help provide basic information and data for life science research, pharmacology, and pharmacokinetics for drug molecules.^10,11^

This research intends to use a variety of spectroscopy methods to study the interaction mechanism of pepsin–DFX system and the effect of DFX on pepsin structure, and initially investigate the effects of DFX on pepsin activity. Moreover, this study aims to use molecular docking and molecular dynamics (MD) simulation calculation methods to obtain the binding model of drug and protein that is most consistent with the actual situation and to study the effects of DFX on the secondary structure and active site of pepsin based on the binding model.

## 2 Materials and methods

### 2.1 Materials and stock solution preparation

Deferasirox (DFX, 98%) was purchased from 3B Pharmachem (Wuhan) International Co., Ltd. Pepsin (98–99%) and bovine hemoglobin solution (99%) were obtained from Sigma-Aldrich Chemical Company (St. Louis, USA). Anhydrous citric, trisodium citrate (dihydrate), absolute ethanol, trichloroacetic acid, and folin-phenol were purchased from Kelon (Chengdu) chemical reagent factory. All reagents are of analytical grade. The water used throughout the experiment was ultrapure water.

Citric acid–sodium citrate buffer at 0.20 mol L^−1^ (pH = 2.0) was prepared. Pepsin stock solution (1.6 × 10^−4^ mol L^−1^) and bovine hemoglobin solution stock solution (0.5 wt%) were prepared in citric acid–sodium citrate buffer and stored at 4 °C in the dark. DOX (1.6 × 10^−5^ mol L^−1^) was prepared with absolute ethanol. Trichloroacetic acid mother liquor (10 wt%) was prepared in citric acid–sodium citrate buffer.

### 2.2 Experimental method

#### 2.2.1 Fluorescence quenching spectra measurements

Steady-state fluorescence quenching spectra were collected using a Cary Eclipse Fluorescence Spectrophotometer (Varian, CA, USA) equipped with 1.0 cm quartz cells. Pepsin–DFX series solutions were prepared in a 5 mL volumetric flask. The final concentration of pepsin was fixed at 1.6 × 10^−5^ mol L^−1^, and the final concentration of DFX was 0, 4.0, 8.0, 12.0, 16.0, 20.0, and 24.0 × 10^−6^ mol L^−1^. The solvent was citric acid–sodium citrate buffer (pH = 2.0). A plurality of the above-mentioned pepsin–DFX series solutions were arranged and were subjected to fluorescence spectroscopy after being incubated for 30 min at 298 K, 304 K, and 310 K. The test conditions were as follows: excitation wavelength 280 nm, emission wavelength measurement range 300–500 nm, excitation sipe width 10 nm, and emission sipe width 10 nm.

#### 2.2.2 Fluorescence lifetime measurements

Fluorescence lifetime measurement in singlet state was executed *via* the time-correlated single-photon counting technique with a Horiba Jobin Yvon FluoroLog-TCSPC spectrofluorometer (HORIBA, Les Ulis, France) at room temperature. The concentration of pepsin was fixed at 1.6 × 10^−5^ mol L^−1^, and pepsin–DOX complex solutions (molar ratios of pepsin to DOX = 1 : 0, 1 : 1, and 1 : 2) were studied. The test conditions were excitation wavelength 280 nm and emission wavelength 345 nm.

#### 2.2.3 Synchronous fluorescence measurements

The same pepsin–DFX series solution as in Section 2.2.1 was prepared and incubated at 298 K for 30 min. The test conditions were as follows. The difference between the emission wavelength and the excitation wavelength were Δ*λ* = 60 nm and Δ*λ* = 15 nm, respectively. The wavelength test range was 200 to 400 nm. The excitation slit width was 10 nm. The emission slit width was 10 nm.

#### 2.2.4 Three-dimensional fluorescence measurements

The concentration of pepsin was fixed at 1.6 × 10^−5^ mol L^−1^, and the solution with a pepsin to DFX molar ratio of 1 : 0 and 1 : 1 was studied. After 3 min of reaction at 298 K, the three-dimensional fluorescence of the two solutions was determined. The test conditions were as follows: excitation wavelength 200–400 nm, emission wavelength 200–400 nm, excitation slit width 10 nm, emission nip width 10 nm, and scanning every 5 nm.

#### 2.2.5 CD spectra measurements

Circular dichroism (CD) spectra were obtained from automatic recording spectrophotometer (Model 400, AVIV, USA) equipped with Peltier temperature control unit in a cell with a path length of 10 mm at 298 K. The concentration of pepsin was fixed at 1.6 × 10^−5^ mol L^−1^, and a solution with a pepsin to DFX molar ratio of 1 : 0, 1 : 1, and 1 : 2 was investigated. After reacting for 30 min at 298 K, the CD spectra of the three solutions were determined. To rule out the interference of citric acid on pepsin circular dichroism, the dilution solvent was deionized water. The scanning wavelength was at 180–260 nm, and the average was obtained by measuring three times.

#### 2.2.6 Enzyme activity measurements

The enzymatic activity of pepsin was tested using the modified Anson method. The same pepsin–DFX series solution as in Section 2.2.1 was prepared and incubated at 310 K for 20 min. Then, 2 mL of bovine hemoglobin solution (0.5 wt%) was added. After 20 min of reaction, 2 mL of trichloroacetic acid (10 wt%) was added to terminate the reaction. The mixed solution was kept at 310 K for 10 min, and then centrifuged at 12 000 rpm for 20 min. The supernatant (1 mL) was added to 1 mL of NaOH solution (4.0 mol L^−1^) and 1 mL of folin-phenol reagent, and the absorbance (OD_660_) of the solution at 660 nm was measured by an ultraviolet spectrophotometer, after being kept at 310 K in a water bath for 15 min. The relative activity of pepsin can be calculated by the following formula:^12^

1 Inhibition rate (%) = (OD_660blank_ − OD_660sample_)/OD_660blank_ × 100

### 2.3 Computational methods

#### 2.3.1 Molecular docking

Molecular docking simulation was performed on the FlexX^13^ docking module built into the LeadIT drug screening platform for the binding model analysis of DFX and pepsin. The DFX molecular 3D structure used was calculated from PubChem (PubChem CID: 5493381), and the pepsin crystal structure was downloaded from the RCSB Protein Database (PDB: 5PEP). The DFX molecule and the pepsin molecule were separately optimized prior to the docking calculation to ensure that the molecule entered the proper protonation state. The calculation was performed using the global docking method, and the docking results were analyzed using the LigPlot program.^14^

#### 2.3.2 MD simulation

In this study, the molecular dynamics (MD) simulation study of the pepsin–DFX interaction system was carried out using YASARA v17.4.17 software.^15^ The molecular force field selected was the AMBER14 force field,^16^ and the local charge number of each atom of DFX was calculated using AM1-BCC^17^ model. The most optimal molecular docking conformation was applied for further analysis in MD simulation. The initial structure was placed in a square water box with a length, width, and height of 100.04 Å under periodic boundary conditions. The pH was set to 2.0 and the temperature was set to 298 K. Sodium and chloride ions were then added to the system to keep it electrically neutral. The simulation was performed using the MD macro (md run) preset in the YASARA software. The van der Waals force threshold was 8.0 Å, and the long-range electrostatic interaction was calculated using the Particle Mesh Ewald (PME) method. Multiple integration steps were used in the calculation. The intramolecular force was 1.25 fs, the intermolecular force was 2.5 fs, and the trajectory was saved every 100 ps.

## 3 Results and discussion

### 3.1 Interaction mechanism analysis

#### 3.1.1 Fluorescence quenching and mechanism

**Fig. 2:**
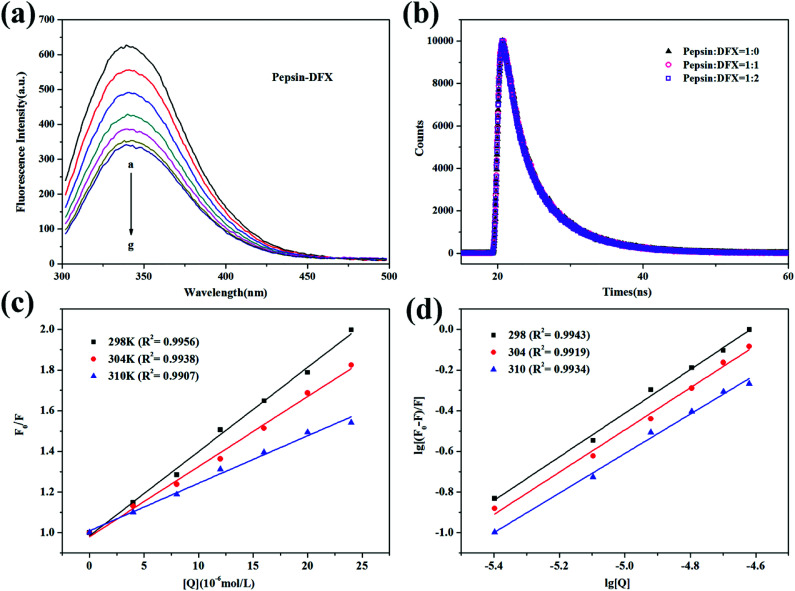
(a) Steady state fluorescence spectra of pepsin–DFX system at 298 K (DFX concentrations from a to g were 0.0, 4.0, 8.0, 12.0, 16.0, 20.0, and 24.0 × 10^−6^ mol L^−1^, respectively. Pepsin concentrations were all 16.0 × 10^−6^ mol L^−1^). (b) Time–resolved fluorescence decay curves of pepsin–DFX system at 298 K. (c) Stern–Volmer plots for the fluorescence quenching of pepsin–DFX system at different temperatures. (d) Double logarithm curves for the fluorescence quenching of pepsin–DFX system at different temperatures.

The pepsin molecule has endogenous fluorescence due to the inclusion of five Trp residues and thirteen Tyr residues.^18^ After the addition of DFX with increasing content in pepsin solution, the fluorescence intensity gradually decreased [Fig. 2(a)]. This fluorescence quenching indicated that the pepsin molecule interacted with the DFX molecule in solution.

The occurrence of fluorescence quenching may be attributed to different quenching mechanisms. Common types of quenching are static and dynamic.^19^ In this article, the more authoritative fluorescence lifetime method was used to judge the mechanism of interaction between DFX and pepsin. As shown in Fig. 2(b), the time-resolved fluorescence spectrum of pepsin solution was almost unchanged before and after the addition of DFX, and the tail-fitting method was further utilized for data analysis. The fitting results were evaluated with *χ*^2^. After three fittings, *χ*^2^ ≈ 1, the fitting results were up to standard. The fitting results are listed in Table 1, and the average fluorescence lifetime (*τ*_ave_) was calculated by the following formula:^20^

2 *τ*_ave_ = *α*_1_*τ*_1_ + *α*_2_*τ*_2_ + *α*_3_*τ*_3_

**Table 1:** Fluorescence lifetime (*τ*) of pepsin with different DFX concentrations

[Pepsin] (×10^−5^ mol L^−1^)	[DFX] (×10^−5^ mol L^−1^)	*τ*_1_ (ns)	*τ*_2_ (ns)	*τ*_3_ (ns)	*α*_1_ (%)	*α*_2_ (%)	*α*_3_ (%)	*τ*_ave_ (ns)	*χ* ^2^
1.6	0	1.836	5.719	0.474	16.71	76.01	7.27	4.688	1.052
	1.6	1.709	5.776	0.360	18.64	75.04	6.32	4.675	1.009
	3.2	1.773	5.799	0.425	18.86	74.26	6.88	4.670	1.042

The average fluorescence lifetime (*τ*_0_) of the blank protein was almost the same as the average fluorescence lifetime of the protein after addition of different levels of DFX molecules (excluding instrumental and operational errors). The fluorescence quenching of pepsin by DFX was a static quenching mechanism, and it was impossible to combine quenching with dynamic and static binding.^21^

Moreover, fluorescence quenching data at different temperatures can be used to analyze the quenching mechanism. For the dynamic annihilation mechanism, the annihilation process conforms to the dynamic annihilation Stern–Volmer eqn (3). The temperature rises, the molecular motion accelerates, more collision annihilation occurs, the annihilation rate increases, and the *K*_D_ increases. For the static quenching mechanism, the quenching process follows the static quenching Stern–Volmer eqn (4). The temperature rises, thereby resulting in a decrease in the stability of the ground state complex. The degree of association of the quencher-fluorescent molecule decreases. The decrease in *K*_SV_ is calculated as follows:^22^

3 *F*_0_/*F* = 1 + *K*_q_*τ*_0_[Q] = 1 + *K*_D_[Q]

4 *F*_0_/*F* = 1 + *K*_SV_[Q]

where *F*_0_ and *τ*_0_ represent the fluorescence intensity and average fluorescence lifetime of the blank fluorescent molecule, respectively. *F* represents the fluorescence intensity of the fluorescent molecule after the addition of the quencher molecule. [Q] represents the concentration of the quencher molecule. *K*_q_ is the quenching rate constant. *K*_D_ is the dynamic quenching constant, and *K*_SV_ is the association constant of the quencher-phosphor.

Fig. 2(c) shows the Stern–Volmer curve of the pepsin–DFX system at 298 K, 304 K, and 310 K, that is, the linear fit of *F*_0_/*F* to DFX molecular concentration [Q]. As the temperature increases, the slope of the Stern–Volmer fitting line decreases, thereby indicating that the fluorescence between the DFX and pepsin molecules was due to the formation of a complex.^23^ The association constants *K*_SV_ were listed in Table 2. In addition, assuming that the annihilation mechanism was a dynamic annihilation, *K*_q_ = 8.835 × 10^12^ L mol^−1^ s^−1^ in the calculated 298 K, which contradicted the dynamic quenching *K*_q_ maximum value of 2 × 10^10^ L mol^−1^ s^−1^.^24^ Again, the principle of fluorescence quenching in the DFX–pepsin system was a static quenching mechanism. Based on the fluorescence lifetime and the trend of Stern–Volmer constant *K*_SV_ of fluorescence quenching at different temperatures, it was confirmed that the fluorescence quenching of pepsin by DFX was a static quenching mechanism, that is, the combination of DFX and pepsin forms a ground state complex.

**Table 2:** Stern–Volmer quenching constants, binding parameters, and thermodynamic parameters of the pepsin–DFX system under three temperatures

*T* (K)	*K*_sv_ (10^4^ L mol^−1^)	*K*_a_ (10^4^ L mol^−1^)	*n*	Δ*G* (kJ mol^−1^)	Δ*H* (kJ mol^−1^)	Δ*S* (J mol^−1^ K^−1^)
298	4.142	8.978	1.073	−28.45	−103.28	−251.12
304	3.447	4.998	1.039	−26.94		
310	2.338	1.782	0.972	−25.44		

#### 3.1.2 Binding constant and number of binding sites

For the system of static quenching mechanism, the binding constant and the number of binding sites can be calculated using the double logarithmic eqn (5):^25^

5 lg[(*F*_0_ − *F*)/*F*] = lg *K*_a_ + *n* lg[Q]

where *K*_a_ represents the binding constant, and *n* represents the number of high affinity binding sites. Fig. 2(d) shows the fluorescence quenching double logarithmic curve of the pepsin–DFX system in 298 K, 304 K, and 310 K. The *K*_a_ and *n* values were listed in Table 2. As the temperature increases, *K*_a_ gradually decreases, thereby indicating that the pepsin–DFX ground state composite weakens at high temperature. The values of *n* at the three temperatures were close to 1, indicating that the DFX small molecule binds to a major binding site in the pepsin.

#### 3.1.3 Thermodynamic parameters and binding forces

The formation of ground state complexes between organic small molecules and biomacromolecules mainly depends on non-covalent forces, such as hydrogen bonds, van der Waals forces, and hydrophobic forces.^26^ Using the Van't Hoff eqn (6), the enthalpy change (Δ*H*) and entropy change (Δ*S*) of the interaction between small molecules and biomacromolecules were calculated. The main forces of the two molecules were judged accordingly. The Gibbs–Helmholtz formula (7) was used to calculate the Gibbs free energy change (Δ*G*) of the interaction to determine whether the binding was spontaneous, as follows:^27^

6 ln *K*_a_ = −Δ*H*/*RT* + Δ*S*/*R*

7 Δ*G* = Δ*H* − *T*Δ*S*

where *K*_a_ is the binding constant at the corresponding temperature. Table 2 shows the calculation results of thermodynamic parameters of pepsin–DFX system. Δ*G* < 0 indicated that the binding between pepsin and DFX molecules was spontaneous; Δ*H* < 0, Δ*S* < 0 indicated that the main force of pepsin and DFX molecule binding was the hydrogen bond.^28^

### 3.2 The effects of interaction on the structure of pepsin

#### 3.2.1 Synchronous fluorescence spectroscopy

**Fig. 3:**
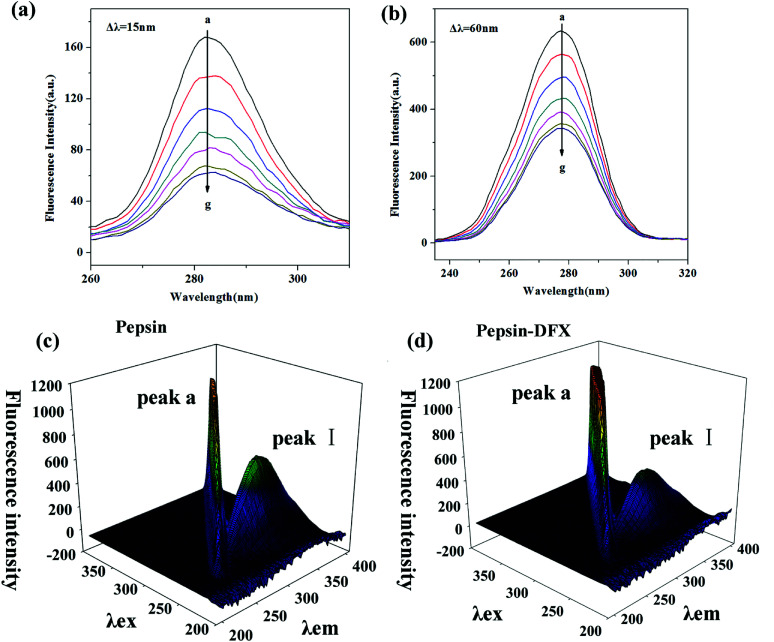
Synchronous fluorescence spectrum of pepsin–DFX system at 298 K, (a) Δ*λ* = 15 nm, (b) Δ*λ* = 60 nm (DFX concentrations from a to g were 0.0, 4.0, 8.0, 12.0, 16.0, 20.0 and 24.0 × 10^−6^ mol L^−1^, respectively. Pepsin concentrations were all 16.0 × 10^−6^ mol L^−1^). (c and d) The 3D fluorescence spectra of pepsin in the absence and presence of DFX.

Synchronous fluorescence can distinguish the overlapping peaks of endogenous fluorescent chromospheres in the ordinary fluorescence spectrum, so that the fluorescence change of a single fluorescent chromophore after the addition of the quencher can be observed.^29^ When set, Δ*λ* = 15, 60 nm, the characteristic fluorescence spectrum of Tyr and Trp in the protein molecule can be determined, respectively. Fig. 3(a) and (b) show the synchronous fluorescence spectra of the pepsin–DFX interaction system. With increasing DFX content in pepsin solution, the fluorescence intensity of both spectra decreased gradually, thereby indicating that DFX quenched the endogenous fluorescence of Trp and Tyr in the pepsin molecule. However, there was no red shift or blue shift on the maximum emission of two spectra, thereby indicating that the interaction between DFX and pepsin had no effect on the microenvironment of the Trp and Tyr residues in the protein.

#### 3.2.2 Three-dimensional (3D) fluorescence spectroscopy

The 3D spectrum of the pepsin solution before and after the addition of DFX is shown in Fig. 3(c) and (d). Peak a is the Rayleigh scattering peak of the protein solution (*λ*_ex_ = *λ*_em_). The addition of DFX molecules enhanced its fluorescence. Peak I (*λ*_ex_ = 280, *λ*_em_ = 340) is the peak caused by the n–π* transition in Trp and Tyr.^30^ The addition of DFX molecules decreased the fluorescence intensity of Peak I of pepsin, but did not show red shift or blue shift (Table 3), indicating that the interaction of DFX and pepsin caused the quenching of Trp and Tyr fluorescence in pepsin but did not change the overall conformation of pepsin.

**Table 3:** Characteristic parameters in the 3D fluorescence spectra of pepsin–DFX system

System	Peak no.	Peak position [*λ*_ex_/*λ*_em_ (nm/nm)]	Intensity
Pepsin	I	280/340	592.596
Pepsin–DFX (1 : 1)	I	280/340	424.454

#### 3.2.3 Circular dichroism (CD) spectra

**Fig. 4:**
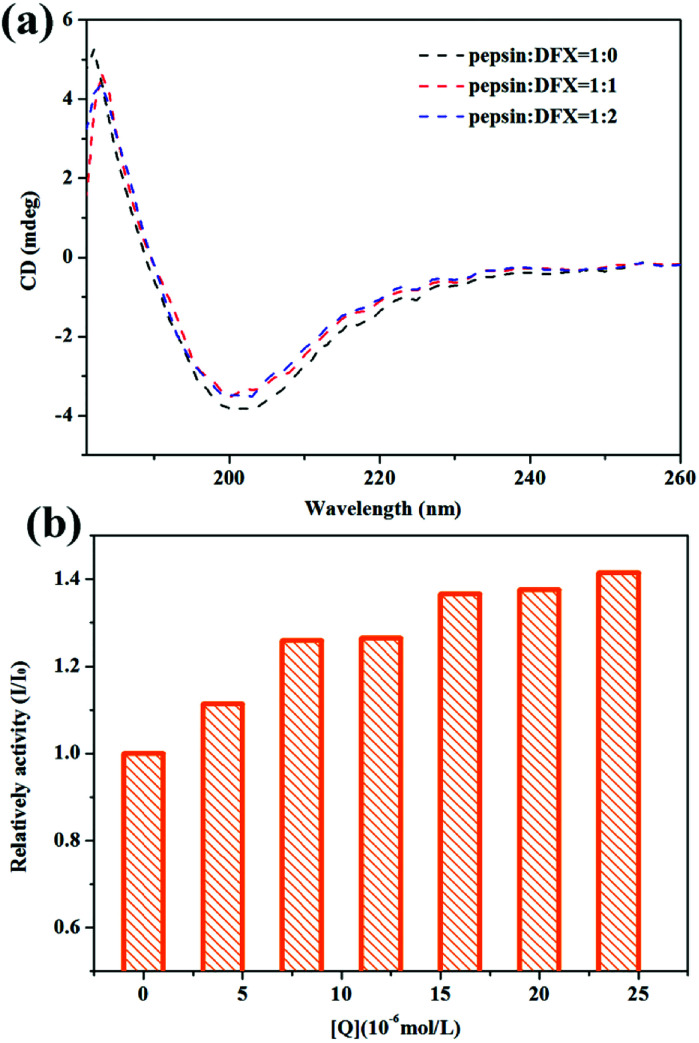
(a) CD spectra of pepsin–DFX system at 298 K. (b) Effect of DFX on pepsin activity *in vitro*. DFX concentrations were 0.0, 4.0, 8.0, 12.0, 16.0, 20.0, and 24.0 × 10^−6^ mol L^−1^, respectively. Pepsin concentrations were all 16.0 × 10^−6^ mol L^−1^.

Fig. 4(a) shows the CD spectra of pepsin solution before and after the addition of DFX. When no DFX molecule was added, the pepsin solution showed a positive peak at 180–190 nm and a negative peak at around 200 nm, which is a typical β-sheet CD spectrum.^31^ From the crystal structure of pepsin, the secondary structure in pepsin is indeed β-folded, thereby indicating that the spatial structure of the protein in pepsin test solution was not destroyed. After adding DFX, the peak shape of CD spectrum of pepsin solution did not change. The peak intensity decreased slightly, thereby indicating that the binding of DFX molecules to pepsin molecules only slightly decreased the content of β-sheets in the protein molecule. The change was minimal for the overall structure of pepsin. The interaction between DFX and pepsin had almost no effect on the structure of the protein.

#### 3.2.4 The effects of interaction on pepsin function

Pepsin is the most important digestive protease in the human body that is capable of hydrolyzing most natural proteins, including plant and animal proteins.^32^ In this study, bovine hemoglobin was used as a catalytic substrate for pepsin to test the effects of the binding of DFX and pepsin on the catalytic activity of pepsin. The results are shown in Fig. 4(b). The catalytic activity of the blank protein was set to 1. After the addition of DFX molecule, the catalytic activity of pepsin was slightly improved, thereby indicating that DFX has an enhanced effect on the activity of pepsin.

### 3.3 Simulation calculation results and discussion

#### 3.3.1 Molecular docking

**Fig. 5:**
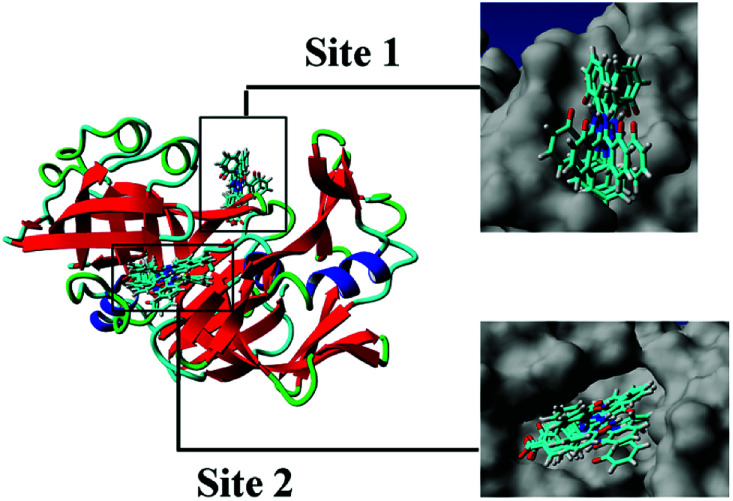
Cluster analysis for the first 10 conformations of DFX binding to pepsin.

Fig. 5 shows the top 10 binding model for FlexX scoring. The binding mode was mainly divided into two categories. The models ranked 1st and 7 to 9 show that DFX bind to the site 1 of pepsin, and the models ranked 2 to 6 and 10 shows that DFX bind to site 2 of pepsin. The binding models with the highest scores in the two major combinations (Rank-1 configuration is Model-1; Rank-2 configuration is Model-2) were closed to analyze the binding force. The results are listed in Table 4. In the Model-1 binding model, DFX was combined with Asn8, Ser161, and Ser163 by four hydrogen bonds, and the total hydrogen bond energy was 76.45 kJ mol^−1^. In addition, DFX was surrounded by Tyr9, Asp159, Asp160, Glu7, and Leu6, which had a hydrophobic interaction of 18.779 kJ mol^−1^ with the DFX molecule [Fig. 7(a)]. The docking results of the Model-2 binding model showed that the main binding force between DFX and pepsin was a hydrophobic interaction of 31.346 kJ mol^−1^, and the amino acids involved in the hydrophobic interaction were as follows: Met290, Tyr189, Tyr75, Ser36, Ile128, Ile73, Thr74, and Gly34. DFX was only linked to Asn37 *via* an O–H⋯N hydrogen bond with a bond energy of 18.18 kJ mol^−1^ [Fig. 8(a)].

**Table 4:** The hydrogen bond energy and hydrophobic interaction energy of two binding models of pepsin–DFX (molecular docking data and MD simulation data)

	Force type	Total energy (kJ mol^−1^)	Hydrogen bond	Bond energy (kJ mol^−1^)
Model-1 docking	Hydrogen bond	76.45	O3⋯O–H (Ser 163)	16.43
			O4⋯O–H (Ser 161)	19.38
			O4⋯H–N (Ser 163)	20.33
			N6–H⋯O (Asn 8)	20.33
	Hydrophobic force	18.779		
Model-1 MD 35 ns	Hydrogen bond	0		
	Hydrophobic force	17.619		
Model-2 docking	Hydrogen bond	18.18	O3⋯H–N (Asn 37)	18.18
	Hydrophobic force	31.346		
Model-2 MD 35 ns	Hydrogen bond	60.10	O3⋯H–O (Ser 35)	22.88
			O4⋯H–N (Ser 36)	16.90
			O4–H⋯O (Ile 128)	20.33
	Hydrophobic force	16.721		

From the analysis of the experimental results in Section 3.1.2, a high affinity binding site was present between DFX and pepsin, and the main force of binding was hydrogen bonding. Compared with the results of the FlexX docking, it seems that the binding mode of Model-1 was more consistent with the experimental results. However, during the molecular docking calculation, the protein was in a static state, and the proteins in the actual solution were dynamic. Only in rare cases could a drug molecule enter a relatively static active site of a protein like a key inserted into a keyhole. In most cases, the identification and binding of drug molecules to proteins is a dynamic process. In this process, changes in protein movement play a crucial role in the binding of most drug molecules. Therefore, further use of molecular dynamics simulation calculations can be used to find the most suitable DFX–pepsin binding mode.

#### 3.3.2 Molecular dynamics simulation process system stability judgment

**Fig. 6:**
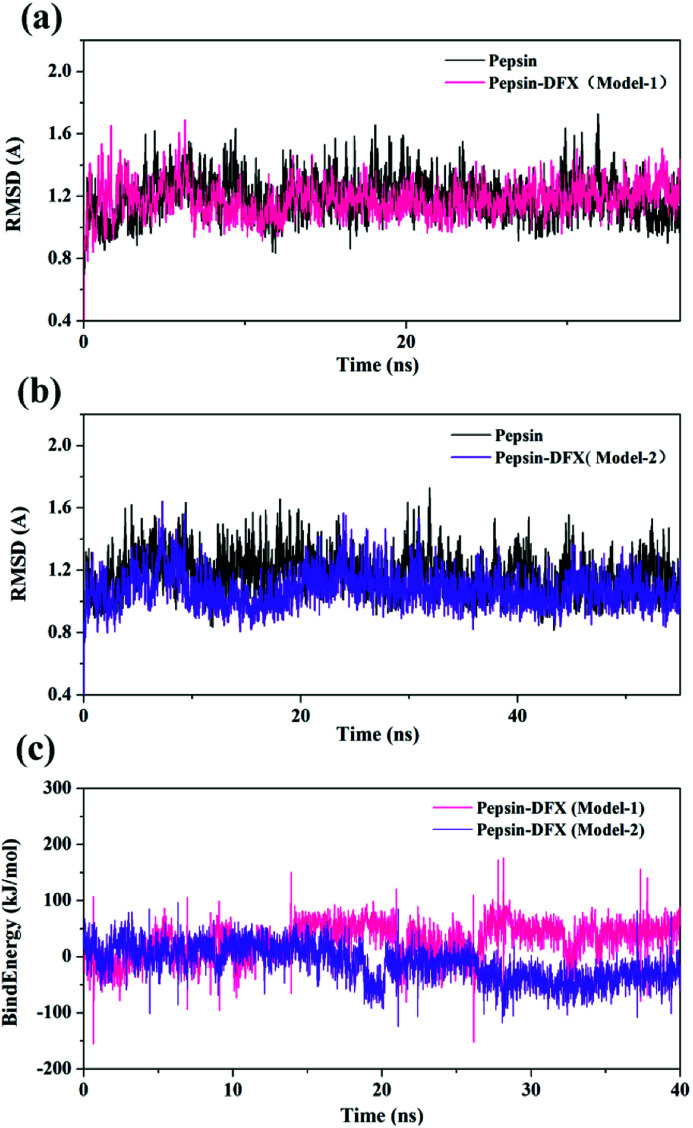
(a and b) RMSD of pepsin and the pepsin–DFX complex (two model). (c) Binding energy of the molecular dynamics simulations of the two pepsin–DFX complex models.

The top 2 scored by the molecular docking results of pepsin–DFX complex structure (Model-1 and Model-2) and the blank pepsin structure were used as the initial conformations for MD simulation calculation. The root mean square deviation (RMSD)^33^*versus* time curve of three system protein skeleton *C*_α_ atoms is shown in Fig. 6(a) and (b). Both the blank pepsin and pepsin–DFX Model-1 systems stabilized after 15 ns, and the pepsin–DFX Model-2 system stabilized after 28 ns. As a result, all systems basically reached equilibrium at 35 ns. Thus, the data at 35 ns were extracted for analysis.

The binding energy of the two binding models during the molecular dynamics simulation was calculated by YASARA and plotted in Fig. 6(c). The binding energy of Model-2 was lower than that of Model-1, thereby indicating that the pepsin–DFX complex was more stable in the binding mode of Model-2. It is preliminarily indicated that the Model-2 structure was more suitable for the actual situation.

#### 3.3.3 The analysis of binding force

**Fig. 7:**
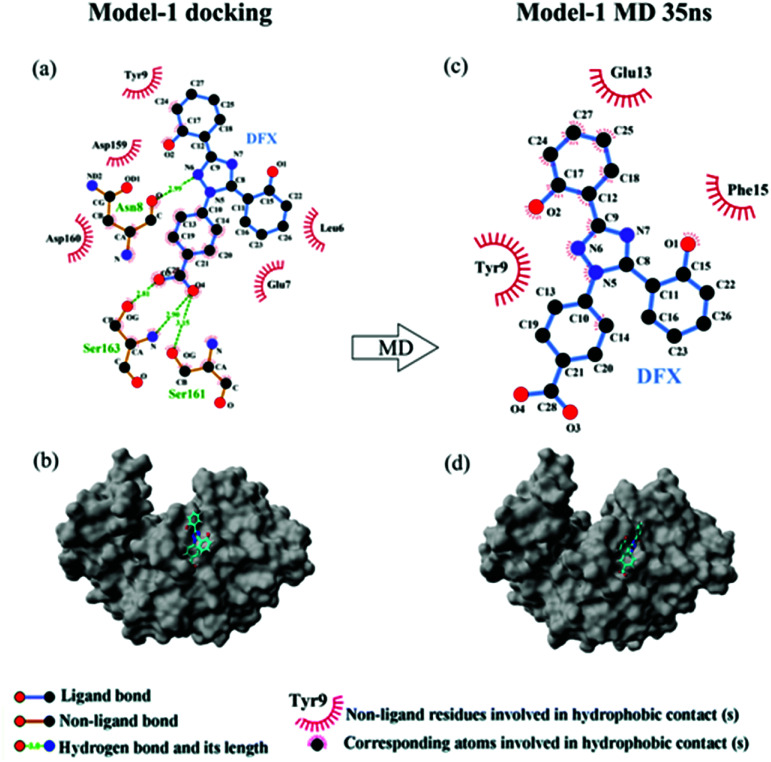
(a) The two-dimensional maps of interactions about pepsin–DFX Model-1 docking result, (b) combined model diagram of pepsin–DFX Model-1 docking result, (c) the two-dimensional maps of interactions about pepsin–DFX Model-1 after MD simulations of 35 ns, (d) combined model diagram of pepsin–DFX Model-1 after MD simulations of 35 ns.

**Fig. 8:**
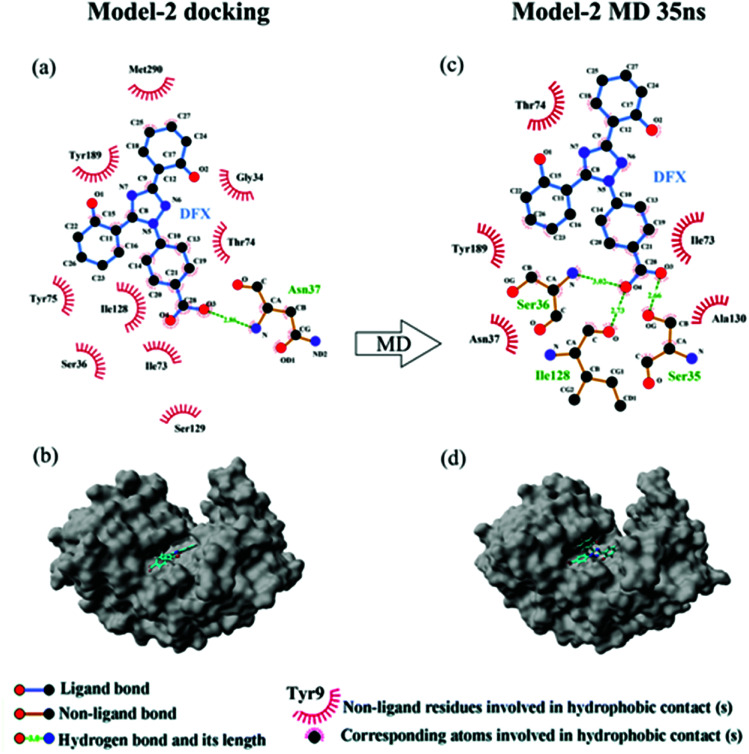
(a) The two-dimensional maps of interactions about pepsin–DFX Model-2 docking result, (b) combined model diagram of pepsin–DFX Model-2 docking result, (c) the two-dimensional maps of interactions about pepsin–DFX Model-2 after MD simulations of 35 ns, (d) combined model diagram of pepsin–DFX Model-2 after MD simulations of 35 ns.

The results of 35 ns are shown in Table 4. At 35 ns, the DFX molecule at Model-1 bound to pepsin mainly through a hydrophobic interaction force of 17.619 kJ mol^−1^. The amino acids involved in hydrophobic interaction were Tyr9, Glu13, and Phe15 [Fig. 7(b)]. At 35 ns, the DFX molecule at the Model-2 mainly bound to pepsin by forming hydrogen bonds (total bond energy of 60.10 kJ mol^−1^) with Ser36, Ile128, and Ser35 amino acids. Moreover, there was a hydrophobic interaction of 16.721 kJ mol^−1^ with Thr74, Tyr189, Asn37, Ala130, and Ile73 [Fig. 8(b)].

**Fig. 9:**
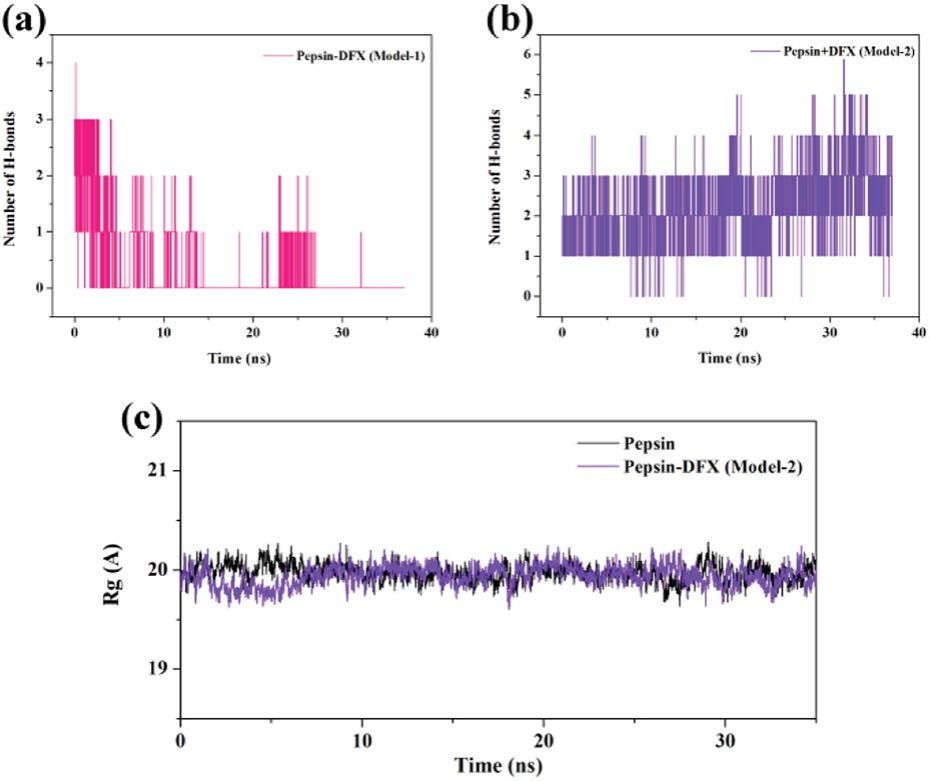
(a and b) The change of H-bond numbers in two kinds of pepsin–DFX complex models during MD calculation. (c) The *R*_g_ of pepsin and the pepsin–DFX complex (Model-2) during MD calculation.

Comparing the results of the docking with the results of MD simulation, the main force of the Model-1 binding mode changed from hydrogen bonding to hydrophobic interaction, whereas the main force of the Model-2 binding mode changed from hydrophobic to hydrogen bond. Among them, the changes in the binding state of DFX to pepsin are shown in Fig. 7 and 8. The change in the number of hydrogen bonds in the MD simulation is shown in Fig. 9(a) and (b). With the dynamic simulation of the protein state, the hydrogen bond in the Model-1 structure gradually broke down until no hydrogen bonding force remained. In the Model-2, the hydrogen bonds were rapidly formed and remained in a state of 3–4 hydrogen bonds. The vibration of amino acid residues in proteins had a great influence on the binding of proteins to drugs. The equilibrium state after molecular dynamics simulation was closer to the actual state than the docking result. Therefore, the Model-2 binding model was a computer model that was more in line with the experimental results.

#### 3.3.4 Effects of interaction on the secondary structure of pepsin

**Fig. 10:**
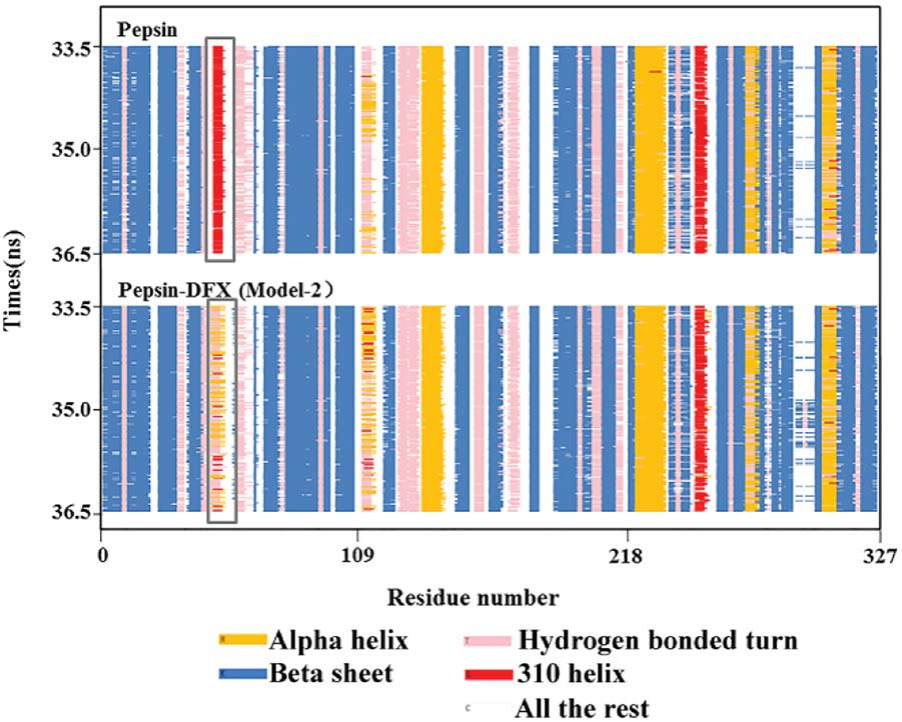
Secondary structure of pepsin.

The Model-2 was more in line with the actual situation. Therefore, and Model-2 was further used to calculate the effects of the DFX on the protein structure. The *R*_g_ values (protein gyration radius)^34^ of the blank pepsin and pepsin–DFX complexes (Model-2) remained stable and similar during the MD simulation calculation [Fig. 9(c)], thereby indicating that DFX did not affect the looseness degree of the pepsin protein structure. Fig. 10 is a schematic diagram of the secondary structure of each amino acid residue in pepsin. The addition of DFX caused the amino acid residue sequence Leu48-Ala49-Cys50-Ser51-Asp52 in pepsin to change from a 310-helix structure to an interconversion structure between hydrogen bond angle and α-helix, although DFX had no effect on the main structure of pepsin. However, the secondary structure of the amino acid fragment near the binding site still produced a significant effect.

#### 3.3.5 Effects of interaction on the conformation of pepsin active site

**Fig. 11:**
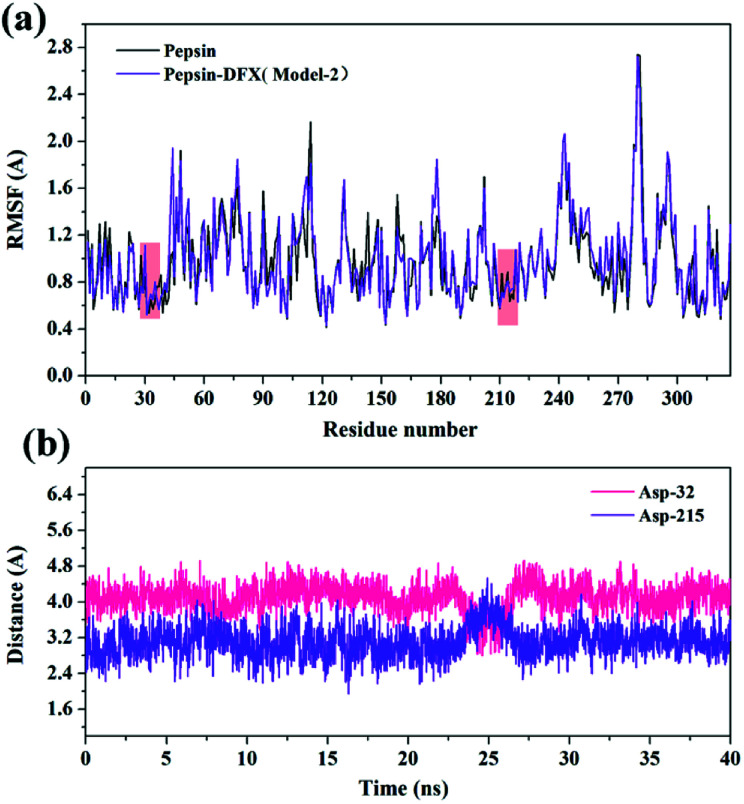
(a) The RMSF of the pepsin and pepsin–DFX complex (Model-2) in MD simulations. (b) The distance between DFX molecules and Asp-32 or Asp-215 in the pepsin–DFX complex during MD calculation.

RMSF^35^ can reflect the degree of fluctuation of the residues in the protein relative to its average position to a certain extent, and it can also be used as a data index to study the dynamic movement of the system. As shown in Fig. 11(a), the addition of DFX had a little effect on the positional fluctuations of active sites Asp-32 and Asp-215. Further calculation of the distance between the DFX molecule and the active sites Asp-32 and Asp-215. The results are shown in Fig. 11(b). The average distance between DFX and Asp-32 was 4.097 Å, and the minimum distance was 2.797 Å. The average distance between DFX and Asp-215 was 3.107 Å, and the minimum distance was 1.944 Å. The distance between DFX and Asp-215 was similar to the bond length of conventional hydrogen bonds. Thus, the dynamic environment of DFX and Asp-215 would affect the electrical environment of Asp-215 residues, which would have a certain effect on the function of 215-Asp.

## 4 Conclusion

The mechanism of this interaction showed that DFX formed a ground state complex with pepsin with only one high affinity binding site for the binding of DFX to pepsin, and the binding process was dominated by hydrogen bonds. According to the calculation of this model, the binding force of DFX and pepsin was mainly hydrogen bonding, and the hydrophobic interaction was supplemented. DFX did not affect the looseness of pepsin protein and the overall secondary structure, but it had a significant effect on the special amino acid residue sequence. The pepsin enzyme activity test showed that the addition of DFX slightly enhanced the activity of pepsin. Combined with the MD results, DFX was close to the pepsin active site Asp-215 (average 3.107 Å, minimum 1.944 Å), which may have affected the electrical environment of Asp-215 residue to enhance the activity of pepsin.

## Conflicts of interest

There are no conflicts to declare.

## Supplementary Material
